# Ischemic stroke induces gut permeability and enhances bacterial translocation leading to sepsis in aged mice

**DOI:** 10.18632/aging.100952

**Published:** 2016-04-25

**Authors:** Joshua Crapser, Rodney Ritzel, Rajkumar Verma, Venugopal R. Venna, Fudong Liu, Anjali Chauhan, Edward Koellhoffer, Anita Patel, Austin Ricker, Kendra Maas, Joerg Graf, Louise D. McCullough

**Affiliations:** ^1^ University of Connecticut Health Center Department of Neuroscience, Farmington, CT 06030, USA; ^2^ University of Michigan Department of Neuroscience, Ann Arbor, MI 48109, USA; ^3^ University of Connecticut Department of Molecular and Cell Biology, Storrs, CT 06269, USA; ^4^ University of Texas Health Science Center at Houston, McGovern Medical School, Houston, TX 77030, USA

**Keywords:** aging, inflammation, infection, experimental stroke, middle cerebral artery occlusion

## Abstract

Aging is an important risk factor for post-stroke infection, which accounts for a large proportion of stroke-associated mortality. Despite this, studies evaluating post-stroke infection rates in aged animal models are limited. In addition, few studies have assessed gut microbes as a potential source of infection following stroke. Therefore we investigated the effects of age and the role of bacterial translocation from the gut in post-stroke infection in young (8-12 weeks) and aged (18-20 months) C57Bl/6 male mice following transient middle cerebral artery occlusion (MCAO) or sham surgery. Gut permeability was examined and peripheral organs were assessed for the presence of gut-derived bacteria following stroke. Furthermore, sickness parameters and components of innate and adaptive immunity were examined. We found that while stroke induced gut permeability and bacterial translocation in both young and aged mice, only young mice were able to resolve infection. Bacterial species seeding peripheral organs also differed between young (*Escherichia*) and aged (*Enterobacter*) mice. Consequently, aged mice developed a septic response marked by persistent and exacerbated hypothermia, weight loss, and immune dysfunction compared to young mice following stroke.

## INTRODUCTION

Stroke is responsible for 1 out of every 20 deaths in the United States [1]. A major contributor to mortality is post-stroke infection [2]. Although aspiration and invasive hospital procedures contribute to infection following stroke, post-stroke immune suppression (PSI) also plays a prominent role [3]. PSI is characterized by lymphopenia and compromised monocyte function, thereby impairing both arms of the immune system leaving the host vulnerable to a variety of pathogens [4].

As in humans, stroke can lead to spontaneous bacterial infections in mice [5] in part due to PSI [5]. Although stroke is primarily a disease of the elderly [1] and aging is one of the most important risk factors for post-stroke infection [7], research on the role of aging in post-stroke infection risk is limited. Aged mice, like their human counterparts, have consistently higher mortality and greater neurological impairment after stroke compared to young mice [8, 9], even though our laboratory and others have shown that aged male rodents have histologically smaller infarcts after experimental stroke than [10]. Indeed, we have shown that aged mice develop smaller infarct volumes and tissue atrophy at 24 hours [9, 11-13] and 30 days [13,14] after middle cerebral artery occlusion as well as less blood-brain barrier permeability [9,15] and leukocyte infiltration [11] but have significantly poorer behavioral recovery than young mice. Peripheral factors such as bacterial infection and immune dysfunction may underlie the poorer post-stroke outcome seen in aged mice, rather than histological damage. Stroke-induced bacterial translocation (BT), the passage of bacteria or bacterial components across the intestinal barrier into extra-intestinal sites [16], has not been explored as a source of infection in aged animal models of stroke and may serve as a potential therapeutic target to improve prognosis.

In this study, we examined intestinal permeability and BT into the mesenteric lymph nodes (MLNs), spleens, lungs and livers of young and aged mice. Both innate and adaptive immune responses occur during bacterial infection, with activation of T cells taking place after an encounter with foreign antigen and simultaneous signaling by innate immune cells. However, the immune response is dramatically altered with age secondary to immune senescence [17]. These age-related impairments in immunity are likely compounded by PSI. We examined post-stroke lymphocyte activation, proliferation and brain infiltration in young and aged mice.

## RESULTS

### Gut permeability increases after stroke and correlates with stroke severity

**Figure 1:**
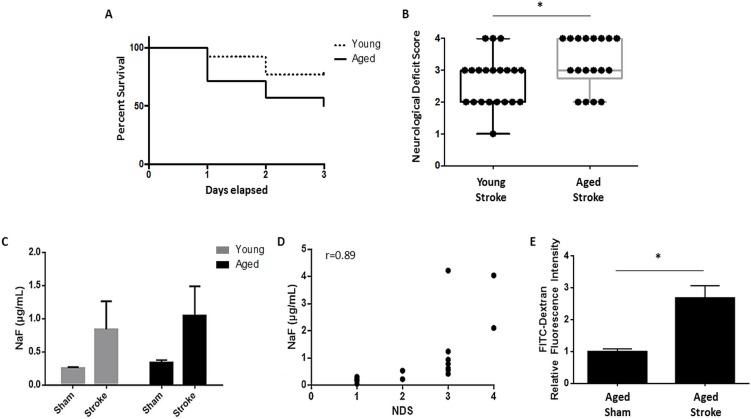
The effects of age on stroke outcome and the effects of stroke on gut permeability (**A**) There was a significant effect of age on mortality 72 hours after 90 minute MCAO as analyzed by Mantel-Cox test of Kaplan-Meier survival curves (p<0.05, n=26-28/group). (**B**) Aged mice had significantly worse NDS than young 72 hours after 90 minute MCAO as measured by Mann-Whitney U test (p=0.05, n=18-21/group). (**C**) There was a significant effect of stroke (F(1,33)=p<0.05, n=9-10/group), but not aging, on intestinal permeability to NaF (MW 376 Da) 72 hours after 90 minute MCAO as measured by 2-way ANOVA. (**D**) The extent of gut permeability to NaF correlated positively with neurological deficit score at this timepoint (r=0.89, p<0.0001, n=18). (**E**) Permeability to the higher molecular weight FITC-Dextran (MW 4 kDa) significantly increased 24 hours after 90 minute MCAO in aged mice in plasma collected 2 hours after gavage (p<0.05, n=4/group). Values in (**B**) are expressed as box plots and values in (**C**, **E**) are expressed as mean ± SEM. Abbreviations: NaF, sodium fluorescein; NDS, neurological deficit score.” *, p≤0.05.

We confirmed that aged mice had significantly smaller infarcts than young mice despite equivalent reductions in cerebral blood flow during ischemia (see supplemental figures). Aged mice had significantly higher mortality (50%) than young mice (23%) 72 hours after a 90 minute MCAO (p<0.05, n=26-28/group; Fig. 1A) while 7 days after 60 minute MCAO aged mice had 50% mortality and young mice had 25% (n=8/group). Aged mice also had significantly worse neurological deficit scores compared to young 72 hours after 90 minute MCAO (p=0.05, n=18-21/group; Fig. 1B). *In vivo* intestinal permeability in mice was determined at this 72 hour timepoint. There was a significant effect of stroke (F(1,33)=p<0.05, n=9-10/group), but not age, on gut permeability to NaF (MW 376 Da) as measured by 2-way ANOVA (Fig. 1C). Interestingly, the extent of gut permeability to NaF at 3 days in all mice was positively correlated with stroke severity as measured by NDS (r=0.89, p<0.0001, n=18; Fig. 1D). Intestinal leakage was further confirmed in aged mice 24 hours after 90 minute MCAO using FITC-Dextran (MW 4 kDa), demonstrating enhanced permeability to comparably higher (10x) molecular weight tracer molecules that occurs earlier following ischemia (p<0.05, n=4/group; Fig. 1E).

### Stroke induces bacterial translocation and organ colonization at 3 days

**Figure 2:**
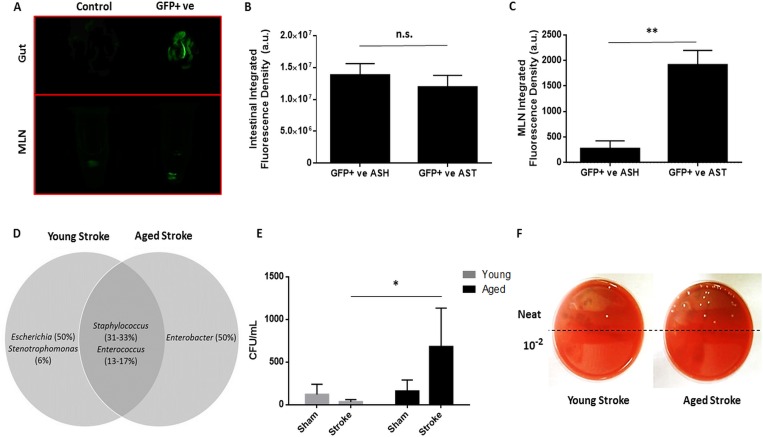
The effects of stroke and age on bacterial translocation 72 hours after 90 minute MCAO and on MLN bacterial burden 7 days after 60 minute MCAO (**A**) Representative image of intestinal and MLN fluorescence in a non-GFP transfected aged sham (“Control”) and in an aged stroke (90min MCAO, 72hr reperfusion) mouse transplanted with GFP+ *E. coli* (“GFP+ ve”). Average fluorescence of controls was subtracted as background from GFP+ aged sham and stroke values. (**B**) While there was no difference in intestinal fluorescence 72 hours after 90 minute stroke or sham surgery (p=0.51, n=4/group) there was a (**C**) significant increase in aged MLN fluorescence following stroke compared to sham values (p<0.01, n=4/group). (**D**) Species of *Staphylococcus* and *Enterococcus* colonized organs from both young and aged mice 72 hours after 90 minute MCAO, bacteria of the genera *Escherichia* and *Enterobacter* were only found in organs from young and aged mice, respectively (n=12-16/group). (**E**) There was a significant effect of age on MLN bacterial burden a week after 60 minute MCAO or sham surgery as measured by a 2-way ANOVA performed on the logarithmically transformed CFU/mL data (F(1,19)=p<0.01, n=4-7/group). Aged mice had significantly greater MLN bacterial burden compared to young at this timepoint following stroke (p<0.05). Data are presented as raw untransformed CFU/mL values. (**F**) Representative image of CFUs grown on blood agar from young and aged stroke MLN homogenates. Values in (**D**) are expressed as the percentage of the total number of successfully identified bacteria that were matched with a particular genus and values in (**B**, **C**, and **E**) are expressed as mean ± SEM. Abbreviations: MLN, mesenteric lymph node; CFU, colony-forming unit; GFP+ ve, microbiome transplanted with GFP-tagged *E. coli*; ASH,aged sham; AST, aged stroke; n.s., not significant; a.u., arbitrary units. *, p≤0.05; **, p<0.01.

Bacterial translocation (BT) primarily occurs through lymphatics, with bacteria from the gut initially draining into the mesenteric lymph nodes [16]. For proof of principle, post-stroke BT was confirmed by transplantation of GFP-transfected E. coli by gavage and enema of aged mice and measuring gut and MLN integrated fluorescence density 72 hours after stroke or sham surgery (Fig. 2A). While there was no difference in intestinal fluorescence (p=0.51, n=4/group; Fig. 2B) there was a significant increase in aged MLN fluorescence following stroke compared to sham values (p<0.01, n=4/group; Fig. 2C) indicating extravasation of GFP-tagged *E. coli*. BT and bacterial burden in the MLNs and other peripheral organs were determined at 3 days after ischemia. Samples plated on blood agar were recorded as either positive or negative for bacterial growth as in [18] (Table 1) (n=6-10/group). The range of bacterial burden found in these organs was also recorded, where bacterial burden was measured as CFUs grown overnight per mL of plated tissue homogenate for each sample. Very little growth was found in young sham mice. Only 1 out of 7 MLN samples tested positive, while no growth was detected in any other organ. Aged sham mice displayed higher BT rates, with 12.5% to 37.5% of samples of each organ type testing positive. Stroke exacerbated BT to the MLNs and increased colonization of other extraintestinal organs. Three days after ischemia, growth was detected in 71.4% of MLNs, 40% of spleens, 80% of livers and 30% of lung samples from young mice, and 85.7% of MLNs, 85.7% of spleens, 100% of livers and 71.4% of lung samples from aged. While high bacterial burden was detected in the MLNs and spleens of both young and aged mice after stroke (reaching levels above 1,000 CFU/mL), similarly extensive bacterial colonization was found only in the livers and lungs of aged stroke mice and not in those from young.

**Table 1:** The frequency of organ samples testing positive for bacteria 72 hours after 90 minute MCAO or sham surgery and the range of bacterial burden in these organs

	Young Sham		Young Stroke		Aged Sham		Aged Stroke	
	(%)	CFU/mL	(%)	CFU/mL	(%)	CFU/mL	(%)	CFU/mL
MLN	14.3	0 – 30	71.4	0 – 1,082	37.5	0 – 10	85.7	0 – >2,000
Spleen	0	0	40	0 – >2,000	12.5	0 – 2	85.7	0 – 1,484
Liver	0	0	80	0 – 36	25	0 – 4	100	12 – >2,000
Lung	0	0	30	0 – 50	12.5	0 – 4	71.4	0 – >2,000

Bacteria were sequenced and entered into a NCBI BLAST search against the 16S rRNA sequence database. *Staphylococcus* and *Enterococcus* species colonized organs in both young and aged mice after stroke. Interestingly, bacteria from the genus *Escherichia* were identified but were unique to organs from young mice after ischemia, and species of *Enterobacter* were only found in organs from aged mice, with about half of the successfully identified bacterial samples from each respective age group matching in sequence to these two genera (Fig. 2D).

### Aging impairs the ability to clear post-stroke infections at 7 days

To assess residual bacterial burden and BT at later time points, MLNs were harvested from young and aged mice 7 days after 60 minute MCAO or sham surgery and plated on blood agar. There was a significant effect of aging (F(1,19)=p<0.01) and a trend towards an interaction between aging and stroke (F(1,19)=p=0.079) on mesenteric bacterial burden 7 days post-stroke or sham surgery as analyzed by 2-way ANOVA on the log-transformed CFU/mL data (n=4-7/group; Fig. 2E). Importantly, there was a significant increase in MLN bacterial burden in aged mice compared to young a week after stroke (p<0.05). All MLN samples from aged stroke mice had burdens greater than 150 CFU/mL, while the majority (57.1%) of MLN samples from young stroke mice had no bacterial burden. The median CFU/mL for aged stroke mice MLNs was 255, while it was 0 for young stroke mice. This difference in bacterial burden is illustrated by a representative image of CFUs on blood agar from harvested MLNs (Fig. 2F).

### Aged mice develop sustained sepsis following stroke

**Figure 3:**
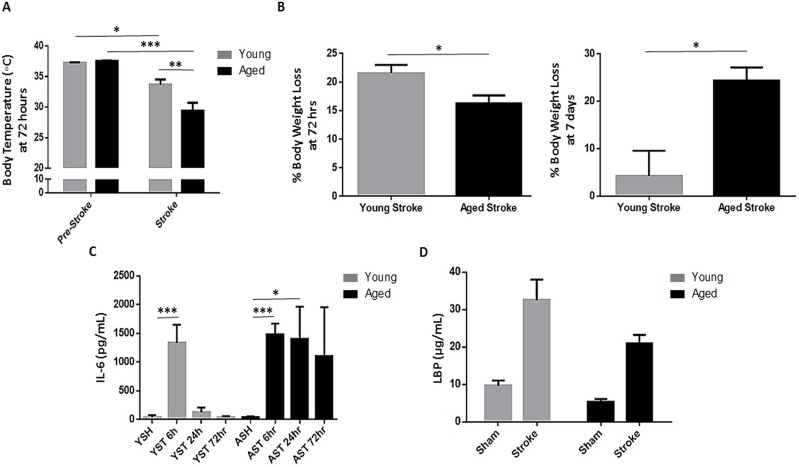
The effects of age and stroke on body temperature, body weight, IL-6 and LBP levels (**A**) There was a significant effect of stroke (F(1,40)=p<0.0001) and aging (F(1,40)=p<0.05) on body temperature 72 hours after 90 minute MCAO as well as an interaction between stroke and aging (F(1,40)=p<0.01, n=11/group) as measured by 2-way ANOVA. Body temperature decreased significantly after stroke in young (p<0.05) and aged (p<0.0001) mice, but aged mice had more severe hypothermia (p<0.01). (**B**) Young mice lost a significantly greater proportion of their initial body weight compared to aged 72 hours after 90 minute MCAO (p<0.05, n=15-19/group), however aged mice sustained significantly more weight loss than young at 7 days after 60 minute MCAO (p<0.05, n=4-7/group), as young mice largely recovered their initial weight by this time. (**C**) There was a significant effect of stroke on IL-6 levels measured 6 hours after 90 minute MCAO in both young (p<0.001) and aged (p<0.0001) mice, returning to sham levels by 24 hours in young mice but remaining elevated in aged at this timepoint (p<0.05) with a similar trend at 72 hours in aged mice (n=5-8/group). (**D**) Both stroke (F(1,36)=p<0.0001) and aging (F(1,36)=p<0.05) had a significant effect on serum LBP levels 72 hours after 90 minute MCAO as analyzed by 2-way ANOVA (n=8-15/group). Values are expressed as mean ± SEM. Abbreviations: IL-6, interleukin-6; A/YSH, aged/young sham; A/YST, aged/young stroke; LBP, lipopolysaccharide-binding protein. *, p≤0.05; **, p<0.01; ***, p<0.001.

Body temperature and weight loss differed after ischemia in young and aged mice. There was a significant effect of stroke (F(1,40)=p<0.0001) and aging (F(1,40)=p<0.05) on body temperature 72 hours after 90 minute MCAO as well as an interaction between stroke and aging (F(1,40)=p<0.01, n=11/group; Fig. 3A). While a significant stroke-induced decrease in body temperature was seen in both young (p<0.05) and aged (p<0.0001) mice, this hypothermia was exacerbated in aged mice (p<0.01). Young mice lost a significantly greater percentage of their pre-stroke body weight compared to aged at 3 days (p<0.05, n=15-19/group), but this trend was reversed at day 7 following a 60 minute MCAO when young mice largely returned to pre-stroke weight but aged mice continued to show weight loss (p<0.05, n=4-7/group; Fig. 3B).

Interleukin-6 (IL-6) is a marker of sepsis severity and mortality [19]. Serum levels of IL-6 were determined by ELISA in young and aged mice 6, 24 and 72 hours after 90 minute MCAO or sham surgery. IL-6 rose significantly at 6 hours after stroke in both young (p<0.001) and aged (p<0.0001) mice compared to sham values. However, while IL-6 dropped to sham levels by 24 hours post-stroke in young mice, it remained increased in aged mice at 24 hours (p<0.05) with a similar trend at 72 hours compared to sham (n=5-8/group; Fig. 3C).

Among the acute phase proteins produced in the liver in response to IL-6 signaling is lipopolysaccharide-binding protein (LBP). This protein binds lipopolysaccharide (LPS) and stimulates an immune response by presenting it to immune cells and facilitating the activation of toll-like receptor 4 complex [20]. Serum LBP levels in young and aged mice 72 hours after a 90 minute stroke or sham surgery showed a significant effect of both stroke (F(1,36)=p<0.0001) and aging (F(1,36)=p<0.05, n=8-15/group; Fig. 3D).

### The lymphocyte response is dysregulated in aged mice following stroke

**Figure 4:**
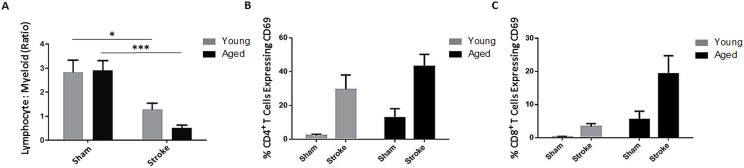
The effects of age and stroke on peripheral lymphocytes 72 hours after 90 minute MCAO (**A**) There was an effect of stroke (F(1,46)=p<0.0001) on the ratio of circulating lymphocytes to myeloid cells, a measure of lymphopenia, as analyzed by 2-way ANOVA (n=10-15/group). This ratio was significantly reduced in young (p<0.05) and aged (p<0.0001) mice following stroke. (**B**) There was a significant effect of stroke (F(1,14)=p<0.005, n=3-6/group) but not age on the proportion of circulating CD69^+^ CD4^+^ T cells as analyzed by 2-way ANOVA. (**C**) The proportion of circulating CD8^+^ T cells expressing CD69 was affected by both stroke (F(1,14)=p=0.05) and age (F(1,14)=p<0.05) via 2-way ANOVA (n=3-6/group). Values are expressed as mean ± SEM. *, p≤0.05; ***, p<0.001.

After finding innate immune differences following stroke in young and aged mice, we investigated the adaptive response to injury. Blood was taken from young and aged mice 72 hours after 90 minute MCAO or sham surgery and the ratio of circulating lymphocytes (CD45^+^CD11b^−^) to myeloid cells (CD45^+^CD11b^+^) as a measure of lymphopenia was determined by flow cytometry. There was a significant effect of stroke (F(1,46)=p<0.0001) on this measure of lymphopenia as analyzed by 2-way ANOVA, and this ratio was significantly reduced in young (p<0.05) and aged (p<0.0001) mice following MCAO compared to sham values, indicating the occurrence of post-stroke lymphopenia in both age groups (n=10-15/group; Fig. 4A). Furthermore, as excessive activation of T cells can be detrimental not only in stroke [21] but can also contribute to poor outcomes in sepsis [22], the activation of circulating CD4^+^ and CD8^+^ T cells concurrent with the observed lymphopenia at the 72 hour timepoint was assessed by expression of CD69, an early marker of T lymphocyte activation, via flow cytometry. ANOVA revealed a significant effect of stroke (F(1,14)=p<0.005), but not aging on the proportion of circulating CD4^+^ T cells expressing CD69 (n=3-6/group; Fig. 4B). The percentage of blood CD8^+^ T cells expressing CD69 was significantly affected by both stroke (F(1,14)=p=0.05) and aging (F(1,14) =p<0.05, n=3-6/group; Fig. 4C).

**Figure 5:**
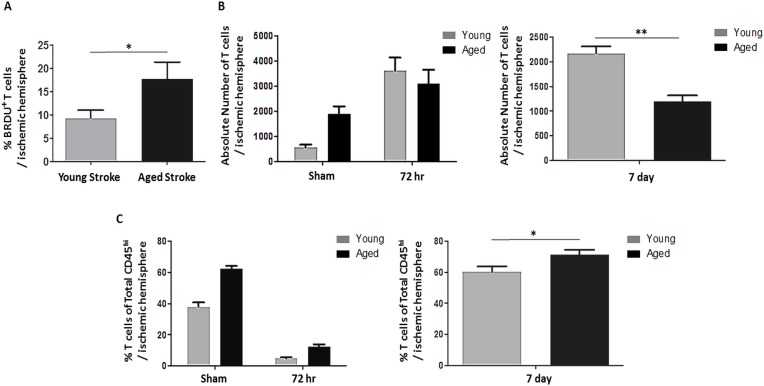
The effects of age and stroke on post-stroke lymphocyte infiltration into the brain (**A**) A significantly larger proportion of T cells infiltrating into the ischemic hemisphere 72 hours after 90 minute MCAO were BrdU-positive in aged mice compared to young (p<0.05)(n=4-6/group). (**B**) There was a significant effect of age (F(1,16)=p<0.001) on the number of T cells infiltrating into the brain 72 hours after 90 minute MCAO as measured by 2-way ANOVA, with significantly less T cells infiltrating into the aged ischemic hemisphere 7 days after 60 minute MCAO as assessed by Student's *t* test (p<0.01, n=4-10/group). (**C**) There was an effect of age (F(1,16)=p<0.0001), stroke (F(1,16)=p<0.0001) and an interaction between age and stroke (F(1,16)=p=0.0001) on the number of T cells (CD3+) as a proportion of all leukocytes infiltrating into the brain (CD45^hi^ cells in the brain) 72 hours after 90 minute MCAO as measured by 2-way ANOVA, with T cells making up a significantly greater proportion of infiltrating leukocytes in aged mice 7 days after 60 minute MCAO as assessed by Student's *t* test (p=0.05, n=4-10/group). Values are expressed as mean ± SEM. *, p≤0.05; **, p<0.01.

In order to track activated T cells in the periphery, we injected BrdU into young and aged mice following MCAO [23]. A significantly larger proportion of CD3*+* T cells infiltrating into the ischemic hemisphere 72 hours after stroke were BrdU-positive in the aged brain compared to the young (p<0.05, n=4-6/group; Fig. 5A). To further characterize infiltrating T cells, we quantified both the absolute number of T cells as well as the proportion of all infiltrating leukocytes that were T cells by flow cytometry at 72 hours after 90 minute MCAO or sham surgery as well as 7 days after a 60 minute MCAO. There was a significant effect of age (F(1,16)=p<0.001) on the number of CD3*+* T cells infiltrating into the brain at 72 hours after stroke as measured by two-way ANOVA, with significantly less T cells infiltrating into the aged ischemic hemisphere 7 days after 60 minute MCAO (p<0.01, n=4-10/group; Fig. 5B) likely due to the smaller infarct seen in aged mice. However, there was an effect of age (F(1,16)=p<0.0001), stroke (F(1,16)=p<0.0001) and an interaction between age and stroke (F(1,16)=p=0.0001) on the number of T cells as a proportion of all leukocytes infiltrating into the brain 72 hours after 90 minute MCAO as measured by 2-way ANOVA, with T cells making up a significantly greater proportion of infiltrating leukocytes in aged mice 7 days after 60 minute MCAO (p=0.05, n=4-10/group; Fig. 5C).

## DISCUSSION

Stroke is commonly complicated by infections that contribute to high rates of morbidity and mortality. An important risk factor for post-stroke infection is advanced age [7]. While this same phenomenon occurs in murine models of ischemic stroke [5], the contribution of the gastrointestinal tract to post-stroke infections has only been investigated in a few studies [18, 24]. Furthermore, the specific role of bacterial translocation in aged mice after stroke has not been studied, although aging increases the prevalence of bacterial translocation in the absence of stroke [25].

Aged mice had higher mortality and more severe neurological impairment than young mice 3 days after ischemic insult. This exacerbated impairment in aged mice is consistent with what we have previously found at 24 hours [9, 11]. Furthermore, aged mice retain deficits compared to sham out to 30 days following ischemia [14], and have persistent deficits compared to young mice on cognitive tests (unpublished data) and on motor tests as documented by others [26, 27]. Stroke severity is an independent predictor of post-stroke infection [28] and spontaneous infections develop in mice following ischemic stroke, especially in subjects with immune deficiency as we hypothesized would occur in aged mice [5, 29]. Interestingly, we found that not only did stroke induce gut permeability at 72 hours of reperfusion, but also that the severity of the stroke was tightly correlated with the extent of permeability. The finding of increased permeability to the higher molecular weight tracer FITC-Dextran at 24 hours provides further evidence that permeability occurs early after stroke and likely contributes to later neurological deficits and higher mortality seen in aged mice. Concurrent with increased permeability was the translocation of bacteria to the MLNs, which does not normally occur in healthy mice, as well as to other peripheral organs. Organs from aged mice tested positive for bacteria more frequently than those from young and this was exacerbated by stroke. Additionally, all organ groups examined in aged mice at 3 days reached relatively high levels of bacterial burden following stroke (>1,000 CFU/mL) while only MLNs and spleens from young mice reached similar degrees of colonization, indicating better containment of bacterial infection in the young.

Although some overlap was seen in the species of bacteria identified in the MLNs and other organs 3 days after stroke, species from the genus *Escherichia* were only found in organs from young mice while *Enterobacter* species were only identified in aged. Important to note is that while we never detected bacteria from the genus Escherichia in the MLNs of these aged mice, we did detect GFP+ E. coli in the MLNs of aged mice following their transplantation into the microbiome (Figure 2A). It should be emphasized that the microbiome of these transplanted mice is artificial inasmuch as it likely contains an unusually high abundance of E. coli, and therefore shouldn't be taken as representative of what bacterial species actually leak out of the intestines following stroke. Rather, the GFP+ E. coli transplantation experiments serve only as proof-of-principal for stroke-induced BT, whereas the samples that were cultured and sequenced should more accurately indicate the identity of extravasating microbes.

Several recent studies report significant age-related changes in gut flora populations [30, 31] and that microbiota from aged individuals is rich in endotoxin-producing gram-negative bacteria such as *Bacteroides* and *Gammaproteobacteria*, which include bacteria of the genera *Escherichia* and *Enterobacter* [32]. However, these genera of bacteria alone are not considered signatures of a young or aged microbiome, and further work from our lab has found that while normal young and aged gut microbiomes differ greatly at the genus level, the ratio of bacteria we report here are not proportionally representative of the overall composition for their respective age group (unpublished data). This indicates that the unique post-stroke translocation of these bacteria bears translational significance, especially since bacteria from both genera have been associated with sepsis [33] and lead to similar mortality rates during bacteremia [34]. It should be recognized that the culturing techniques used in this study were selective for aerobic bacteria. Aerobic bacteria have been shown to make up a considerable proportion of translocating microbes [35], nonetheless the diversity of microbes translocating from the gastrointestinal tract is likely greater than observed here as anaerobic bacteria were not directly assessed.

Evidence of increased gut permeability has been seen in other neurological disorders including multiple sclerosis. Adoptive transfer of autoreactive encephali-togenic T cells disrupts the intestinal barrier, high-lighting a key role for T cells in this process [36]. Gut inflammation, mediated by the release of IL-17, TNFa and IFNy by T helper and antigen-presenting cells results in epithelial tight junction disassembly and subsequent permeability. As a result, intestinal bacteria and their products can cross the gut barrier and activate circulating lymphocytes, increasing their potential to cause injury when recruited to the ischemic brain. T cells play a role in immune defense against bacterial translocation, as their loss results in increased bacterial extravasation from the gut [37]. The phagocytic activity of mononuclear cells is also in part regulated through the secretion of IFNy by Type 1 helper (Th1) T cells [38]. As stroke results in a specific depletion of lymphocytes it is likely that stroke-induced immuno-suppression, in combination with exacerbated intestinal permeability, permits the translocation of bacteria from the gut to MLNs. The failure of aged mice to clear bacteria is likely due in large part to the combined effects of post-stroke lymphopenia and the reduction in the naïve T cell pool and T cell receptor diversity that occurs with immunosenescence [17].

Sepsis is defined as a systemic inflammatory response to infection, manifesting in symptoms such as hypothermia and tachycardia. Aging is a risk factor for sepsis incidence and mortality [39] and the gut is a potential source of sepsis-causing infections [40]. The incidence of severe sepsis increases 100-fold with age and the mortality rates more than double [41]. Serum Interleukin-6 levels rise during sepsis and serve as a prognostic marker of disease outcome [19]. Aged mice had elevated serum levels of IL-6 for 24 hours following stroke, when levels had returned to baseline in young mice. Interestingly, aged mice had lower post-stroke levels of LBP than young in our study. As high levels of LBP can actually dampen the inflammatory response to LPS during sepsis, and injection of LPB rescues septic mice [42], the inability of aged mice to mount an effective LBP response may be a primary mechanism behind their susceptibility to sepsis.

In addition to innate immune activation following stroke, we found alterations in adaptive immunity. Lymphopenia was observed in both young and aged mice three days after MCAO. At the same time that this lymphopenia occurred, we observed extensive activation of circulating cytotoxic T cells and helper T cells in young and aged mice 3 days after MCAO as indicated by CD69 expression. CD8^+^ cytotoxic T cells underwent more extensive activation in aged mice compared to young 3 days after MCAO. Interestingly, T cells are activated during sepsis, and extensive activation can increase disease severity and can negatively influence sepsis outcome [43]. Cell surface expression of CD69 is induced early following T cell receptor (TCR) activation via antigen presentation or by cytokine stimulation [44], and the presence of circulating CD69^+^ T cells after stroke suggests that T cell activation had already occurred in peripheral lymphoid tissue. We found a significantly increased percentage of BRDU-positive T cells in the ischemic brain of aged mice compared to their young counterparts, implying that antigen-driven T cell expansion is exacerbated with age after stroke. We speculate that T cells in the MLNs are activated following stroke due to the leakage of commensal and dietary antigens from the gut. This activation would then be exaggerated in aged mice due to their failure to clear extravasating bacteria, consistent with our data.

It is important to note that fewer T cells migrate into the aged ischemic brain compared to young. This is consistent with previous data showing similarly mitigated leukocyte infiltration [11] concurrent with the smaller infarcts aged mice develop after stroke. Despite this overall reduction in infiltration into the ischemic hemisphere in aged mice, we show that T cells make up a significantly greater proportion of the overall leukocytic infiltrate relative to young. As a result, the composition of the leukocytes migrating into the young and aged brain following ischemic stroke is different. Furthermore, aged T cells are more extensively activated and undergo greater proliferation in response to stroke than young. We do not however try to suggest that any one particular cell type is responsible for or driving this dysregulated immune response, as it is likely all cells play an important role. Significant defects or reductions in either T cell or B cell compartments (or NK/NK T cells for that matter) can have profound implications on stroke outcome and often worsen post-stroke immune suppression [5, 45]. Nonetheless the quality of inflammation in both central and peripheral environments may better reflect the severity of injury seen in aged individuals rather than the quantity of infiltrating leukocytes.

One limitation of this study is the use of different occlusion times for more chronic survival studies. Given the high mortality aged mice experience in the days following 90 minute MCAO (50% in this study), we chose a 60 minute MCAO for 7 day timepoints to enhance survival. Consequently, 90 minute occlusions were used to study changes within the first 3 days of reperfusion, while 60 minute occlusions were used to asses effects at 7 days. This approach allowed us to determine age-related differences at a given timepoint after stroke surgery while efficiently increasing survival at 7 days of reperfusion and reducing the number of mice needed to complete the study. Importantly, comparisons were made between age groups at each timepoint, each of which had an identical occlusion time.

In summary, intestinal permeability and bacterial translocation is induced following ischemic stroke in both young and aged male mice. The failure of aged mice to resolve bacterial translocation and inflammation, marked by excessive activation of both the innate and the adaptive immune systems, results in prolonged sepsis. This, in addition to the age-related alteration of leukocyte trafficking patterns into the ischemic brain, may contribute to the increased morbidity and mortality seen in aged mice compared to young following stroke, despite the former's smaller infarcts. Future studies are needed to determine the mechanism by which a distant, sterile injury such as stroke can induce bacterial leakage from the gut.

## METHODS

### Experimental animals

All animal protocols were approved by the University's Institutional Animal Care and Use Committee and were performed in accordance with National Institutes of Health and RIGOR guidelines. Young (8-12 weeks) and aged (18-20 months) C57Bl/6 male mice were acclimated in pairs to the housing conditions in an ambient temperature and humidity controlled vivarium with a 12-12 hour day-night cycle for one month. Mice had free access to food and water *ad libitum* and after sham or stroke surgery were provided with wet mash for food.

### Stroke surgery

Mice were randomly assigned to sham or stroke surgery groups. Transient focal cerebral ischemia was induced by right MCAO for 90 or 60 minutes under Isoflurane anesthesia as described previously [46] followed by 72 hours or 7 days of reperfusion. A 60 minute MCAO was used to assess changes at 7 days in order to minimize mortality. A 0.21mm silicone coated suture was used to perform MCAO in young mice and a 0.23mm suture was used for aged mice [11] resulting in equivalent cerebral blood flow reductions during ischemia as measured by laser Doppler flowmetry [9] and laser speckle flowmetry [15]. Rectal temperature and body weight were recorded immediately prior to MCAO and at 3 or 7 days after stroke. Rectal temperatures were monitored during surgery and maintained at ∼37°C with an automated heating pad.

### Neurological deficit

Neurological deficit scores (NDS) were determined immediately following reperfusion as well as 72 hours after MCAO using an established scoring system as described previously by a blinded observer [11].

### *In vivo* intestinal permeability

Intestinal permeability in stroke and sham mice was assessed by oral gavage of the fluorescent tracer sodium fluorescein (NaF, MW 376 Da) 3 days after MCAO surgery. Mice were fasted overnight and gavaged with 10ug/g body weight NaF while under anesthesia [36]. Blood was collected 2 hours after oral gavage and centrifuged at 3000rpm at 4°C for 20 minutes. Concentrations of NaF in plasma were determined in 96-microwell plates by measuring fluorescence at an excitation of 400nm and emission of 516nm and using standard concentrations of NaF for comparison.

In a separate cohort, aged mice were administered 0.5 ml of FITC-dextran in saline solution by gavage 24 hours after 90 minute stroke or sham surgery. 2 hours following oral gavage, blood was collected and centrifuged and plasma was analyzed in a 96-microwell plate at an excitation of 470nm and emission of 520nm to determine mean fluorescence intensity [47].

### Bacterial translocation

To determine the occurrence of bacterial translocation, stroke and sham mice were euthanized and transcardially perfused with phosphate-buffered saline (PBS). Mesenteric lymph nodes (MLNs), spleens, and one lobe each from livers and lungs were removed and homogenized in 1% saponin, and the homogenate was incubated on ice for one hour. Homogenates were then plated on blood agar and incubated at 37°C for 16 hours and colony-forming units (CFUs) were examined. Bacterial burden was expressed as CFUs per mL of saponin homogenate. To determine identity, bacteria colonies were collected, suspended in PBS and pelleted. DNA was extracted from cell pellets using the MoBio Power Mag kit (MO BIO Laboratories, Inc.) and genomic DNA was determined as described in supplementary methods.

In order to further visualize and quantify bacteria extravasating from the gut into the MLNs in aged mice, GFP-expressing plasmids (ABM Inc.) were transfected into *Escherichia coli* by standard methods. Transformed *E. coli* were grown by overnight incubation at 37°C in LB medium. GFP-expressing *E. coli* colonies were selected by visual confirmation under fluorescence microscopy. Mice were administered with 0.5 ml of GFP-expressing *E. coli* suspension/day by gavage and 0.5 ml solution of GFP-expressing *E. coli* suspension was administered into the colon by enema [48]. 24 hours after transplantation, aged mice underwent 90 minute stroke or sham surgery. At 72 hours post-stroke, MLNs and intestines were collected and images were captured using a fluorescence microscope. Data is presented as integrated fluo-rescence density minus the average integrated fluorescence density of control (non-GFP transfected, aged sham) MLNs or intestines.

### ELISA

Serum was collected from stroke and sham mice at time of euthanasia and lipopolysaccharide-binding protein (LBP) levels were determined by ELISA (Abnova) following the manufacturer's instructions. Serum interleukin-6 (IL-6) levels were determined using multiplex (Millipore).

### Flow cytometry

In order to assess cellular changes with flow cytometry, young and aged stroke and sham mice were euthanized and blood and brain was assessed by flow cytometry as in [49]; see supplementary methods for details and gating strategy. To label proliferating CD3*+* T cells, 5-Bromo-2′-deoxyuridine (BrdU) was injected intraperitoneally 12, 24 and 48 hours after stroke, as described in [49].

### Statistics

Data are presented as mean ± standard error of the mean (SEM) except NDS data, which is presented as a box plot. Groups were compared with Student's *t* test, Mann-Whitney U test, or 2-way analysis of variance (ANOVA) with Tukey or Bonferroni corrections for multiple comparisons where appropriate (GraphPad Prism Software Inc.). In order to run a 2-way ANOVA on the nonparametric CFU data at 7 days post-stroke, the data was transformed by adding 10 (the lowest nonzero value in our data set) to all CFU/mL values and taking the log base 10 of the sum. Mortality was analyzed by generating Kaplan-Meier survival curves and performing a Mantel-Cox log-rank test. Correlational analysis of NDS and gut permeability was determined by generating a Spearman's correlation coefficient. Statistical significance was set at p≤0.05. Experiments and data analyses were performed blinded to surgical conditions and age.

## SUPPLEMENTAL DATA


