# Supplementary material for: Ischemic stroke induces gut permeability and enhances bacterial translocation leading to sepsis in aged mice
Source: Aging (Albany NY). 2016 Apr 25;8(5):1049–60. doi: 10.18632/aging.100952 (PMC4931853; doi:10.18632/aging.100952)
Supplement: Supplementary file 1 [file aging-08-1049-s001.pdf]

SUPPLEMENTAL DATA

METHODS

Infarct analysis and laser Doppler flowmetry. A separate cohort of young and aged mice underwent 60 minute MCAO surgery following the protocol outlined in the primary manuscript. During MCAO surgery, cerebral blood flow to the ischemic hemisphere was measured with laser Doppler flowmetry (Moor Instruments) as described previously [1]. At 7 days of reperfusion, mice were euthanized and transcardially perfused with PBS followed by 4% paraformaldehyde. Brains were extracted, post-fixed for 24 hours and placed in 30% sucrose for cryoprotection. Using a freezing microtome, brains were cut into 30- μm free-floating sections and every eighth slice was stained with cresyl violet for analysis of ischemic damage. These stained sections were imaged and infarct volumes were measured with SigmaScan Pro 5 (SPSS Inc.). A blinded investigator calculated infarct volumes as a percentage of the intact contralateral hemisphere.

Bacterial translocation and sequencing. Genomic DNA was used as template DNA for PCR amplification of the 16S rRNA gene. The reaction included Phusion High Fidelity DNA Polymerase (25 mM TAPS-HCl (pH 9.3 @ 25°C), 50 mM KCl, 2 mM $MgCl^2$, 1 mM b-mercaptoethanol, 200 µM dNTPs including [^{3}H]-dTTP and 15 nM primed M13 DNA), bovine serum albumin (0.06 % final), and 10 uM bacterial specific primers 27F (AGAGTTTGATCMTGGCTCAG) and 1391R (GACGGGCGGTGTGTRCA). The PCR reaction was incubated in a thermocycler (Eppendorf Mastercycler Pro 384 and Control Panel) for 3.5 min at 95.0°C then for 30 cycles of 30 s at 95.0°C, 30 s at 50.0°C and 90 s at 72.0°C, followed by final extension as 72.0°C for 10 minutes. PCR products were cleaned with the QIAGEN GeneRead Size Selection Kit before sequencing from 27F on an ABI 3500 Genetic Analyzer using BigDye Terminator v1.1 (Life Technologies). Sequences were run in a NCBI BLAST search against the 16S rRNA sequence database, and matches were determined as having ≥ 97% identity.

Flow cytometry. Blood was drawn via cardiac puncture with heparinized needles, and mice were subsequently transcardially perfused with PBS. Red blood cells were lysed with three consecutive 10-minute incubations with Tris-ammonium chloride (Stem Cell Technologies). Brains were harvested and the brainstem, cerebellum and olfactory bulbs were removed and brains were split into left and right hemispheres and processed as described previously [2]. Blood and brain leukocytes were washed and blocked with mouse Fc Block (eBioscience) prior to staining with primary antibody-conjugated flourophores: CD45-eF450, CD11b-APCeF780, CD4-APC, MHCII-FITC (eBioscience), and CD3-BV510, CD8-PerCpCy5.5, CD69-PE (BioLegend). For live/dead discrimination, a fixable viability dye, carboxylic acid succinimidyl ester (CASE-AF350, Invitrogen), was diluted at 1:300 from a working stock of 0.3mg/mL. Cells were briefly fixed in 2% paraformaldehyde (PFA). Data were acquired on a LSRII using FACsDiva 6.0 (BD Biosciences) and analyzed using FlowJo (Treestar Inc.). No less than 100,000 events were recorded for each sample. Peripheral T cells were identified as $CD45^+CD11b^-CD3^+$, peripheral lymphocytes as $CD45^+CD11b^-$, peripheral myeloid cells as $CD45^+CD11b^+$, and peripheral leukocytes and T cells infiltrating into the brain as $CD45^{hi}$ and $CD45^{hi}CD11b^-CD3^+$, respectively, relative to resident microglia, which express CD45 at intermediate levels ($CD45^{int}$). Cell type-matched fluorescence minus one (FMO) controls were used to determine the positivity of each antibody.

FIGURES

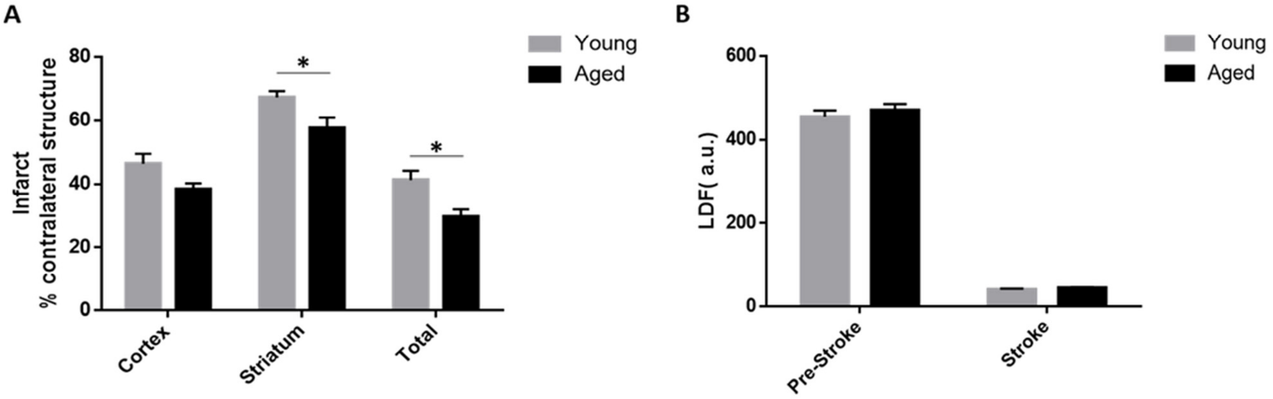

Figure S1. The effects of age on infarct size 7 days after 60 minute MCAO. (**A**) There was a significant effect of age on infarct size as analyzed by 2-way ANOVA ($p<0.0001$, n=8/group) with aged mice having significantly smaller striatal ($p<0.05$) and total ($p<0.05$) infarcts compared to young following post-hoc Bonferroni corrections for multiple comparisons. (**B**) Laser Doppler flowmetry of the same cohort of mice revealed no significant differences in cerebral blood flow reduction during MCAO between young and aged mice as measured by 2-way ANOVA ($p>0.05$, n=8/group). Values are expressed as mean ± SEM. Abbreviations: LDF, laser Doppler flowmetry; a.u., arbitrary units. *, $p\leq0.05$.

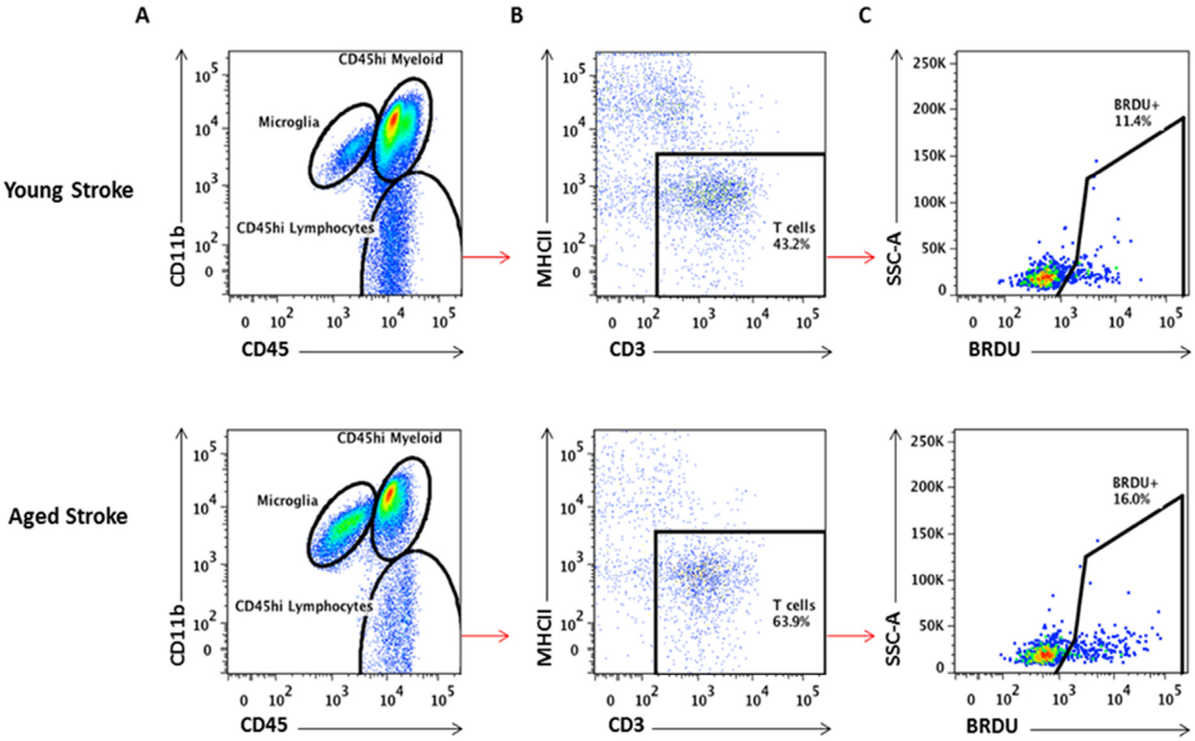

Figure S2. Brain flow cytometry gating strategy using representative flow plots of young and aged mouse brains 72 hours after 90 minute MCAO. (**A**) Brain immune cells were gated on CD45 and CD11b expression in order to differentiate resident microglia ($CD45^{int}CD11b^{+}$), infiltrating myeloid cells ($CD45^{hi}CD11b^{+}$) and infiltrating lymphocytes ($CD45^{hi}CD11b^{-}$). (**B**) Infiltrating lymphocytes from (**A**) were then gated on MHCII and CD3 in order to identify T cells ($CD45^{hi}CD11b^{-}CD3^{+}$). (**C**) Infiltrating T cells from (**B**) were then gated on side scatter (granularity) and BrdU in order to identify BrdU+ T cells, which could then be compared between young and aged mice. Abbreviations: SSC-A, side scatter.

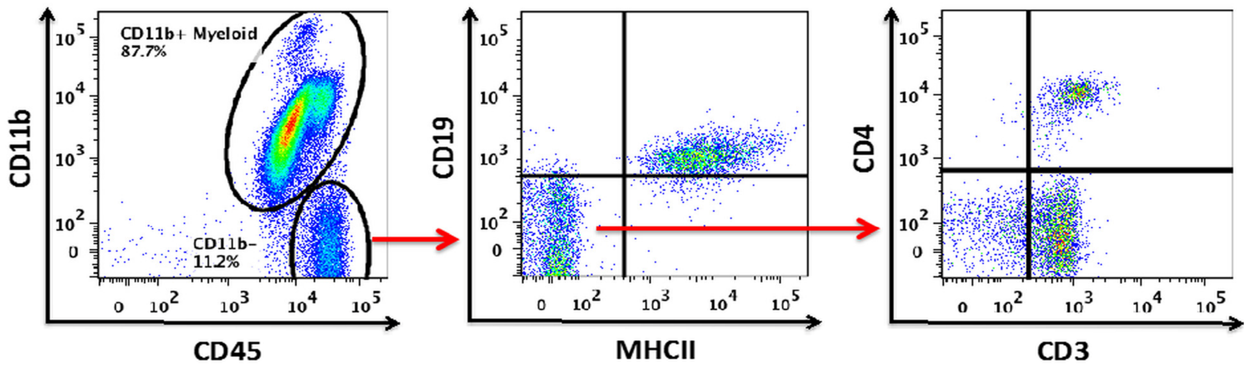

Figure S3. Identification of blood leukocyte populations in aged mice after stroke. (**A**) Blood leukocytes from aged stroke mice were gated on CD45 and CD11b expression in order to differentiate myeloid cells ($CD45^{hi}CD11b+$ from lymphocytes ($CD45^{hi}CD11b^{-}$). (**B**) Lymphocytes from (A) were then gated on MHCII and CD19 in order to identify B cells ($CD45^{hi}CD11b^{-}CD19+MHCII+$) and T cells ($CD45^{hi}CD11b-CD19-MHCII-$). (**C**) T cells from (B) were then gated on CD3 expression to confirm T cell identity and that the majority of $CD45^{hi}CD11b-$ cells are indeed lymphocytes (B cells or T cells).

REFERENCES

1. McCullough L, Wu L, Haughey N, Liang X, Hand T, Wang Q, Breyer RM, Andreasson K. Neuroprotective function of the PGE2 EP2 receptor in cerebral ischemia. J Neurosci. 2004; 24:257-268.

2. Ritzel RM, Patel AR, Grenier JM, Crapser J, Verma R, Jellison ER, McCullough LD. Functional differences between microglia and monocytes after ischemic stroke. J Neuroinflammation. 2015; 12:106-015-0329-1.
